# Cincho-Quinine

**Published:** 1873-07

**Authors:** 


*Cincho-Quinine.*

We are of the opinion, from statements of medical brethren who have tested the cincho-quinine, that it is a very valuable article, equal to, and for some purposes superior to, the sulphate of quinine. It will be seem from advertisement in this journal that Redwine & Fox keep the article on hand. We hope the profession will try this remedy.

